# Effect of prolonged sitting on dynamic cerebral autoregulation in the anterior and posterior cerebral circulations

**DOI:** 10.1113/EP092178

**Published:** 2024-10-25

**Authors:** Shotaro Saito, Hayato Tsukamoto, Marino Karaki, Narumi Kunimatsu, Shigehiko Ogoh

**Affiliations:** ^1^ Department of Biomedical Engineering Toyo University Saitama Japan; ^2^ Faculty of Sport Sciences Waseda University Saitama Japan; ^3^ Graduate School of Health and Sport Sciences hukyo University Aichi Japan; ^4^ Neurovascular Research Laboratory University of South Wales Pontypridd UK

**Keywords:** cerebral blood flow velocity, middle cerebral artery, posterior cerebral artery, sedentary, transfer function analysis

## Abstract

Individuals who experience prolonged sitting daily are reported to be at risk of developing cerebrovascular disease, which is associated, in part, with attenuation in cerebral blood flow regulation. However, the effect of prolonged sitting on dynamic cerebral autoregulation (dCA), a crucial mechanism of cerebral blood flow regulation, remains unclear. Additionally, cerebrovascular disease occurs heterogeneously within cerebral arteries. The purpose of the present study was to examine the hypothesis that prolonged sitting attenuates dCA in the cerebral circulation heterogeneously. Twelve young, healthy participants were instructed to maintain a seated position for 4 h without moving their lower limbs. Mean arterial pressure and mean blood velocities of the middle cerebral artery (MCA *V*_m_) and the posterior cerebral artery (PCA *V*_m_) were measured continuously throughout the experiment. The dCA was assessed using transfer function analysis (TFA) with mean arterial pressure and either MCA *V*_m_ or PCA *V*_m_. In the MCA, very low‐frequency TFA‐normalized gain decreased significantly during 4 h of prolonged sitting (*P* = 0.029), indicating an improvement rather than attenuation in dCA, despite a significant reduction in MCA *V*_m_ after 4 h of continuous sitting (*P* = 0.039). In the PCA, PCA *V*_m_ remained stable throughout the 4 h sitting period (*P* = 0.923), and all TFA parameters remained unchanged throughout the 4 h of sitting. Contrary to our hypothesis, these results suggest that the dCA in both the MCA and the PCA was well stabilized in healthy young individuals during acute prolonged sitting.

## Abstract (highlights)

- **What is the central question of this study?**
  The effect of prolonged sitting on dynamic cerebral autoregulation, a crucial regulator of cerebral blood flow, especially in the posterior cerebral circulation, remains unclear. We investigated the impact of prolonged sitting on the dynamic cerebral autoregulation in the anterior (middle cerebral artery) and posterior cerebral circulations (posterior cerebral artery) in healthy, young individuals.
- **What is the main finding and its importance?**
  Dynamic cerebral autoregulation in the middle and posterior cerebral arteries was not impaired by prolonged sitting. This finding suggests that the effect of prolonged sitting on dynamic cerebral autoregulation is minimal in healthy, young individuals.

## 1 INTRODUCTION

In recent years, prolonged sitting has become a prevalent lifestyle, contributing to the development of physiological dysfunction in the brain (Kerr & Booth, 2022), including cognitive dysfunction (Falck et al., 2017), a heightened risk of stroke (Kyu et al., 2016) and cerebral atrophy (Arnardottir et al., 2016). However, the mechanism behind development of these brain dysfunctions in sedentary individuals who experience habitually prolonged sitting daily remains unclear. Understanding the physiological mechanisms leading to these brain dysfunctions from sedentary behaviour, particularly prolonged sitting, might be crucial for preventing these issues (Burton, 2000). Therefore, several previous studies have examined the impact of a single prolonged sitting session on cerebral blood velocity (CBV) to identify potential risks associated with the onset of brain dysfunction. These studies reported that uninterrupted sitting for >3.5 h reduces CBV in the anterior cerebral circulation [i.e., middle cerebral artery (MCA)] (Baker et al., 2018; Carter et al., 2018; Wheeler et al., 2019). This finding suggests that inadequate regulation of cerebral blood flow (CBF), which fails to sustain optimal brain function, might contribute to the increased brain dysfunction associated with prolonged sitting, because irregularities in CBV might be linked to dysfunction in CBF regulatory mechanisms (Claassen et al., 2021).

Dynamic cerebral autoregulation (dCA) is a fundamental cerebrovascular function crucial for enabling cerebral vessels to maintain adequate CBF and cerebral circulatory homeostasis against fluctuations in blood pressure (Claassen et al., 2021). Therefore, in addition to examining CBF responses, it is crucial to investigate the impact of dCA to identify the risk of cerebrovascular dysfunction or cerebral disease (Dawson et al., 2003; den Abeelen et al., 2014). Indeed, impairment of dCA in the MCA has been observed, especially in patients with stroke and Alzheimer's disease (Dawson et al., 2003; den Abeelen et al., 2014), establishing it as a recognized indicator of cerebrovascular health (Chen et al., 2014; Dawson et al., 2003; den Abeelen et al., 2014; Xiong et al., 2017). Given this background, several previous studies (Carter et al., 2018, 2021; Maasakkers et al., 2020) have investigated the effect of prolonged sitting on dCA in the MCA. However, these findings are inconsistent and suggest that dCA in the anterior cerebral circulation remains unchanged or is impaired after uninterrupted sitting for >3 h (Carter et al., 2018, 2021; Maasakkers et al., 2020). Therefore, the impact of prolonged sitting on dCA in the anterior cerebral circulation remains a topic of debate. Importantly, these studies have variations in subject age (i.e., older and middle‐aged or young individuals) and sitting conditions (sitting duration; 3, 4 or 6 h) (Carter et al., 2018, 2021; Maasakkers et al., 2020). Considering that CBF is maintained during uninterrupted sitting for <3.5 h and then begins to decrease (Wheeler et al., 2019), along with the fact that ageing reduces cerebral circulation and function (Lu et al., 2011; Salthouse, 2009), differences in these experimental conditions (sitting duration and age of subjects) might contribute to the inconsistencies observed among these previous studies.

In addition to the anterior cerebral circulation, the posterior cerebral circulation [e.g., posterior cerebral artery (PCA)] plays an important role in brain function but responds differently to various physiological conditions (e.g., heat stress, exercise, orthostatic stress, hypoxia) compared with the anterior cerebral circulation (Sato & Sadamoto, 2010; Ogoh et al., 2013b, 2013c, 2015; Sato et al., 2012; Washio et al., 2018). Clinically, most strokes in the macrovascular (MCA and PCA) territory occur heterogeneously in a single vessel and rarely occur simultaneously in separate brain territories and cerebral vessels (Nichols et al., 2021). Therefore, in different areas of the cerebral circulation, it is thought that the cerebral vessels can function and adapt independently (Haubrich et al., 2004; Perko et al., 2011; Skow et al., 2013). Indeed, it has been reported that dCA in the PCA in resting conditions is lower than dCA in the MCA (Haubrich et al., 2004). These differences might be attributable to variations in anatomical and physiological characteristics among different brain regions, including the magnitude of diameter and sympathetic innervation (Edvinsson et al., 1976). Hence, the cerebral vascular function in the anterior cerebral circulation might not directly reflect the overall cerebral circulation or CBF regulatory function, including those in the posterior cerebral circulation. However, no study has focused primarily on the response of the posterior cerebral circulation to prolonged sitting; therefore, the effect of prolonged sitting on the posterior cerebral circulation remains unknown. Relying solely on the evaluation of cerebrovascular function in a single cerebral vessel (e.g., only MCA) might risk overlooking the potential for developing cerebral disease, particularly focal conditions such as stroke, in other cerebral territories, including the posterior cerebral circulation (e.g., PCA). Therefore, it might be important to understand regional differences in the cerebral vascular response during prolonged sitting.

In the present study, we aimed to examine the effects of a 4 h period of prolonged sitting on dCA in both the MCA and PCA in young, healthy participants. To achieve this, we carried out transfer functional analysis (TFA) using spontaneous fluctuations in MAP and CBV, which allows for evaluation of dCA without the need for sitting breaks. It has been suggested that dCA in the posterior cerebral circulation (e.g., dCA in the PCA) is more susceptible to orthostatic stress (Haubrich et al., 2004; Sato et al., 2012) and sitting with eyes open (Nakagawa et al., 2009), in comparison to the anterior cerebral circulation (e.g., dCA in the MCA). Therefore, we hypothesized that prolonged sitting impairs dCA in both the MCA and PCA, with the deterioration of dCA being more pronounced in the PCA.

## 2 MATERIALS AND METHODS

### 2.1 Ethical approval

The protocol was approved by the Institutional Review Board of Toyo University (approval number: TU2022‐025) and complied with the standards outlined in the *Declaration of Helsinki*, except for registration in a database. All participants provided written informed consent before their participation.

### 2.2 Participants

Based on a previous study reporting that prolonged sitting decreased CBV, we estimated sample size by setting the effect size (η^2^) to 0.36, with significance level = 0.05 and power = 0.80. Assuming a within‐participant correlation of 0.5, the effective sample size (*n*) was 12, and we recruited 12 participants.

Overall, 12 healthy young participants with relatively sedentary lifestyles (nine males and three females; mean age, 21 ± 1 years; height, 166 ± 7 cm; weight, 60 ± 12 kg; and body mass index, 22 ± 4 kg/m^2^) were recruited from a university for the present study. The participants in this study were university students, whose daily routines typically involved extended periods of sitting, such as commuting, attending lectures and studying. None of the participants had any known cerebrovascular or cardiovascular disease; they were not taking medications and were non‐smokers. Before the experiment, each participant was required to abstain from caffeine, alcohol, vitamin C supplements and strenuous exercise for 24 h. Moreover, given the potential effects of sleep deprivation and the stress of prolonged sitting, participants were instructed to maintain their normal sleep patterns before the study day to ensure that they arrived at the laboratory well rested on the day of the experiment.

Furthermore, the participants were required to refrain from eating on the experimental day. All female participants (*n* = 3) underwent the experiment during the early follicular phase (days 2–5) of their natural menstrual cycle. The experiments started at 09.00 h and ended at ∼16.30 h. The room temperature was set at 24°C–25°C, and the room light was constant. All participants were instructed to stay awake for ≥2 h before the experiment.

### 2.3 Experimental procedure

Upon arrival at the laboratory, participants were placed in a long sitting position (i.e., legs flat position), and instruments for all measurements were attached. Then, the participants were placed in a sitting position, and the baseline values of cardiovascular and cerebrovascular haemodynamics and dCA were evaluated.

After completing these baseline measurements, participants were given a 15 min break, during which they consumed a light meal (i.e., one banana) and engaged in light stretching exercises (e.g., bending), considering the extended duration of the experiment. If necessary, participants used the restroom for urination and defecation. After the break period, participants were instructed to remain seated for 4 h without moving their lower limbs. Based on previous studies indicating reduced CBF attributable to prolonged sitting (Carter et al., 2018), participants were allowed to engage in cognitively undemanding, desk‐based activities, such as using their upper limbs, watching television or reading a book, until 10 min before the measurement sessions.

Throughout the 4 h of sitting, measurements of cardiovascular and cerebrovascular haemodynamics were taken every hour, and dCA was evaluated. All measurements were conducted while participants were in a sitting position. All participants completed the full 4 h sitting duration without any interruption (e.g., restrooms and dropouts).

### 2.4 Experimental measurement

All measurement variables were measured for 5 min in each measurement session, including baseline. During the sitting period, measurements were taken 15 min before the target sitting time was reached and were completed by the time the target sitting time was achieved. Additionally, during all measurement sessions, participants were instructed to keep their eyes open and to focus on a single point to account for the effects of metabolic activity in the PCA territory (i.e., the visual cortices) (Nakagawa et al., 2009) and to simulate conditions of real‐life sedentary activities where eyes are open.

Heart rate (HR) was measured using a lead II ECG (PhysioFlow PF‐05 Lab1; Manatec Biomedical, Paris, France). Continuous beat‐to‐beat arterial blood pressure (ABP) was monitored using finger photoplethysmography (Finometer Pro; Finapres Medical Systems, Amsterdam, The Netherlands) with a cuff placed on the middle finger of the right hand at heart level. The finometer cuff was attached only during the measurement session. To calibrate the beat‐to‐beat ABP for any fluctuations caused by reattaching the finometer cuff, systolic and diastolic blood pressure values in the brachial artery were measured using automated blood pressure monitoring (Tango +; SunTech Medical, Eynsham, Witney, UK). The end‐tidal partial pressure of carbon dioxide ($P_{\mathrm{ET},CO_{2}}$) was measured breath by breath using an automated gas analyser (AE‐310S; Minato Medical Science, Osaka, Japan). Middle cerebral artery and PCA blood velocity (MCA *V* and PCA *V*) were measured through the right and left temporal window, respectively, by using a transcranial Doppler ultrasonography system (DWL Doppler Box‐X; Compumedics, Singen Germany). The CBV response to carotid compression and visual stimuli confirmed that the Doppler signal was recorded correctly from the MCA and PCA. The transcranial Doppler ultrasonography probe was fixed and held in a measurement position using a headband (elastic headband; DWL) to maintain a constant insonation angle throughout the experiment. The positions of the probe and headband were recorded upon completing the measurement set‐up for MCA *V* and PCA *V*, and adjustments were made if any misalignment occurred. Some MCA *V* and PCA *V* data were excluded owing to a technical issue that prevented proper measurement and adjustment (MCA *V*; 3 h, *n* = 2; 4 h, *n* = 1; and PCA *V* all time points, *n* = 2; and 1 h, *n* = 2).

### 2.5 Data analysis

The ABP waveform, HR, $P_{\mathrm{ET},CO_{2}}$, MCA *V* and PCA *V* were analysed offline using signal processing software (LabChart 8; ADInstruments). For ABP calibration, the average values of systolic and diastolic blood pressures over 30 s from the beat‐to‐beat ABP, required for the automated sphygmomanometer, were converted to match the units of systolic and diastolic blood pressure values from the brachial blood pressure readings. Mean arterial pressure (MAP), mean MCA *V* and mean PCA *V* (MCA *V*_m_ and PCA *V*_m_) were derived from each waveform. The indexes of MCA and PCA cerebrovascular resistance (MCA CVRi and PCA CVRi) were calculated as the MAP divided by MCA *V*_m_ and PCA *V*_m_, respectively. The MCA and PCA cerebrovascular conductance indexes (MCA CVCi and PCA CVCi) were calculated as MCA *V*_m_ and PCA *V*_m_ divided by MAP, respectively. All measurement variables were averaged for 5 min.

### 2.6 Dynamic cerebral autoregulation analysis

The analysis of dCA was performed according to a white paper by the Cerebrovascular Research Network (CARNet) (Panerai et al., 2023). The MAP, MCA *V*_m_ and PCA *V*_m_ were acquired across each cardiac cycle, linearly interpolated, and resampled at 4 Hz for spectral analysis and transfer function analysis (TFA) of dCA. The dCA was computed as the transfer function gain and phase shift between fluctuations in MAP and MCA *V*_m_ or PCA *V*_m_. The transfer function gain and phase shift reflect the relative amplitude and time relationship between changes in MAP and MCA *V*_m_ or PCA *V*_m_ over a specified frequency range. Using the temporal sequences of MAP and MCA *V*_m_ or PCA *V*_m_, frequency‐domain transformations were computed with a fast Fourier transformation algorithm. The transfer function *H*(*f*) between the two signals was calculated as *H*(*f*) = *S_xy_* (*f*)/*S_xx_* (*f*), where *S_xx_* (*f*) is the autospectrum of changes in MAP and *S_xy_* (*f*) is the cross‐spectrum between MAP and MCA *V*_m_ or PCA *V*_m_. The transfer function magnitude |*H*(*f*)| and phase spectrum |Φ(*f*)| were obtained from the real part *H*_R_(*f*) and imaginary part *H*_I_(*f*) of the complex function. Additionally, the transfer function *H*(*f*) was normalized to the mean values of input (*x*) and output (*y*) variables as *H*′(*f*) = [*S_xy_* (*f*)*x*]/[*S_xx_* (*f*)*y*], and the normalized gain (nGain) was calculated as 20 log *H*′(*f*) to provide values in decibels. Spectra were calculated using a 100 s window length with 50% overlap, and smoothing was achieved by the use of a Hanning window. Transfer function phase, nGain and coherence were calculated in the very low‐ (VLF; 0.02–0.07 Hz), low‐ (LF; 0.07–0.20 Hz) and high‐frequency (HF; 0.20–0.30 Hz) ranges, as described previously (Panerai et al., 2023; Watanabe et al., 2022). The phase quantifies the time delay, and nGain quantifies the damping effect, reflecting the relative time and amplitude relationship between changes in MAP and MCA *V*_m_ or PCA *V*_m_ within a specified frequency range (Zhang et al., 1998a). Coherence indicates the fraction of output power (i.e., MCA *V*_m_ or PCA *V*_m_) that is linearly related to input power (i.e., MAP) within the same frequency range (Zhang et al., 1998a). We used the VLF and LF nGain, and phase, which were considered dCA to be the most operan as dCA index based on the previous studies (Fisher et al., 2008; Tsukamoto et al., 2021).

### 2.7 Statistical analysis

All data are presented as the mean ± SD or median (interquartile range) and analysed using statistical software (SPSS Statistics v.27; International Business Machines, Tokyo, Japan). The normality of data distribution was assessed using the Shapiro–Wilk test. Normally distributed data were analysed using one‐way repeated‐measures ANOVA followed by Student's paired *t*‐test with Bonferroni correction, and non‐normally distributed data were analysed using non‐parametric ANOVA (Friedman test) followed by Wilcoxon matched‐pairs tests with Bonferroni correction. In the event of missing values, a linear mixed model was used. A *P*‐value of <0.050 was regarded as significant.

## 3 RESULTS

### 3.1 Cardiorespiratory and cerebrovascular variables

During the 4 h sitting, MAP and $P_{\mathrm{ET},CO_{2}}$ remained unchanged (*P* = 0.429 and *P* = 0.194, respectively; Table 1). In addition, although there was a significant time effect in HR (*P* = 0.026), it did not differ from baseline to each time point during 4 h sitting (baseline vs. 1 h, *P* = 0.246; baseline vs. 2 h, *P* = 1.000; baseline vs. 3 h, *P* = 1.000; baseline vs. 4 h, *P* = 0.104).

**TABLE 1:** Cardiovascular and cerebrovascular haemodynamics during 4 h of sitting.

Parameter	Base	1 h	2 h	3 h	4 h	Main effect of time, *P*‐values
HR (beats/min)	64 ± 6	68 ± 7	67 ± 6	67 ± 9	69 ± 8	0.026
MAP (mmHg)	89 ± 7	88 ± 9	88 ± 10	92 ± 14	90 ± 11	0.429
$P_{\mathrm{ET},CO_{2}}$ (mmHg)	37.2 ± 3.4	38.2 ± 3.1	38.1 ± 2.8	38.5 ± 3.3	37.8 ± 2.6	0.194
MCA CVRi (mmHg/cm/s)	1.40 ± 0.33	1.43 ± 0.36	1.49 ± 0.40	1.49 ± 0.44	1.52 ± 0.31^a^	0.044
PCA CVRi (mmHg/cm/s)	1.91 (1.62–2.63)	1.91 (1.74–2.01)	1.99 (1.71–2.25)	2.12 (1.92–2.37)	2.05 (1.74–2.22)	0.595
MCA CVCi (cm/s/mmHg)	0.75 ± 0.17	0.75 ± 0.19	0.72 ± 0.20	0.72 ± 0.20	0.63 ± 0.24^a^	0.025
PCA CVCi (cm/s/mmHg)	0.51 ± 0.13	0.53 ± 0.13	0.53 ± 0.11	0.50 ± 0.17	0.52 ± 0.13	0.904

*Note*: All values are expressed as the mean ± SD or median (interquartile range).

Abbreviations: HR, heart rate; MAP, mean arterial pressure; MCA CVCi, index of middle cerebral artery cerebrovascular conductance; MCA CVRi, index of middle cerebral artery cerebrovascular resistance; PCA CVCi, index of posterior cerebral artery cerebrovascular conductance; PCA CVRi, index of posterior cerebral artery cerebrovascular resistance; $P_{\mathrm{ET},CO_{2}}$, end‐tidal partial pressure of carbon dioxide.

^a^ *P* < 0.05 versus base.

**FIGURE 1:**
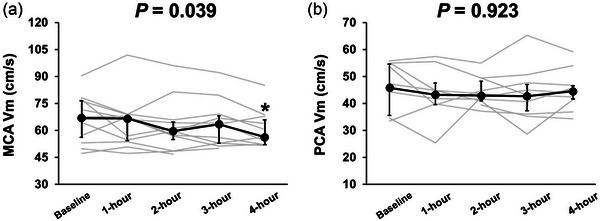
Mean blood velocity of middle cerebral artery (MCA *V*_m_; a) and posterior cerebral artery (PCA *V*_m_; b). All values are shown as the median (interquartile range).

Regarding CBF parameters, during the 4 h sitting there was a significant time effect in MCA *V*_m_, MCA CVRi and MCA CVCi (*P* = 0.039, *P* = 0.044 and *P* = 0.025, respectively; Figure 1; Table 1). Specifically, MCA *V*_m_ and MCA CVCi were significantly decreased (baseline vs. 4 h, *P* = 0.029 and *P* = 0.032, respectively), and MCA CVRi was significantly increased at 4 h from the onset of sitting compared with baseline (baseline vs. 4 h, *P* = 0.035). In contrast, PCA *V*_m_, PCA CVRi and PCA CVCi remained unchanged throughout the 4 h sitting (*P* = 0.923, *P* = 0.595 and *P* = 0.904, respectively).

### 3.2 Power spectrum in the spectral analysis

In the VLF range, the power spectrum of MAP, MCA *V*_m_ and PCA *V*_m_ remained unchanged during the 4 h sitting periods (*P* = 0.695, *P* = 0.097 and *P* = 0.096, respectively; Table 2). Likewise, in the LF range, the power spectrum of MAP, MCA *V*_m_ and PCA *V*_m_ also remained unchanged during the 4 h sitting periods (*P* = 0.702, *P* = 0.057 and *P* = 0.927, respectively).

**TABLE 2:** Spectral power of mean arterial pressure, and mean blood velocities of the middle and posterior cerebral arteries during 4 h of sitting.

Parameter	Base	1 h	2 h	3 h	4 h	Main effect of time, *P*‐values
MAP (mmHg^2^)						
VLF	4.7 (11.4–2.1)	8.6 (14.1–2.8)	7.0 (14.5–4.9)	6.9 (10.9–3.4)	5.4 (8.7–2.5)	0.695
LF	2.0 (1.0–7.6)	4.0 (11.4–2.0)	4.2 (6.3–1.4)	4.2 (9.8–1.9)	4.8 (6.2–2.2)	0.702
MCA *V*_m_ (cm/s)^2^						
VLF	7.9 (10.9–7.0)	8.5 (11.4–5.6)	6.4 (10.2–4.3)	10.9 (13.2–4.8)	3.8 (5.3–2.3)	0.097
LF	4.3 (6.9–2.5)	6.2 (9.4–4.8)	3.8 (6.9–2.5)	5.1 (7.9–3.9)	3.0 (5.3–2.6)	0.057
PCA *V*_m_ (cm/s)^2^						
VLF	4.1 ± 3.1	5.5 ± 2.9	5.0 ± 2.3	7.0 ± 4.4	3.1 ± 2.0	0.096
LF	4.3 ± 4.7	5.2 ± 4.2	4.7 ± 4.9	5.1 ± 4.5	4.8 ± 6.7	0.927

*Note*: All values are expressed as the mean ± SD or median (interquartile range).

Abbreviations: VLF, very low frequency; LF, low frequency; MAP, mean arterial pressure; MCA *V*_m_, mean blood velocity of middle cerebral artery; PCA *V*_m_, mean blood velocity of posterior cerebral artery.

^*^ *P* < 0.05 versus base.

### 3.3 Dynamic cerebral autoregulation in the MCA

In the VLF range, phase and coherence remained unchanged during the 4 h sitting periods (*P* = 0.442 and *P* = 0.218, respectively). There was a significant time effect in nGain (*P* = 0.036), notably, it decreased significantly at 4 h from the onset of sitting [baseline vs. 4 h, 1.3 (0.9–2.0) vs. 0.8 (0.6–1.7) dB, *P* = 0.029]. In the LF range, phase, nGain and coherence were unchanged during the 4 h sitting periods (*P* = 0.454, *P* = 0.547 and *P* = 0.743, respectively).

### 3.4 Dynamic cerebral autoregulation in the PCA

In the VLF range, phase, nGain and coherence remained unchanged during the 4 h sitting periods (*P* = 0.957, *P* = 0.593 and *P* = 0.054, respectively). Likewise, in the LF range, phase, nGain and coherence were unchanged during the 4 h sitting periods (*P* = 0.545, *P* = 0.918 and *P* = 0.758, respectively).

## 4 DISCUSSION

**FIGURE 2:**
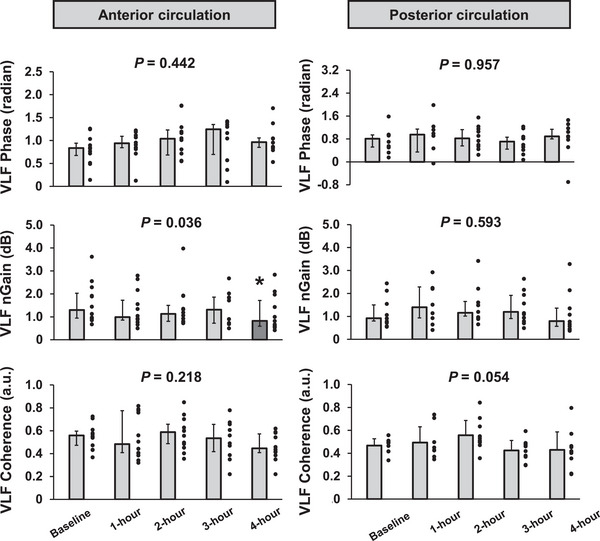
The transfer function phase, normalized gain (nGain) and coherence of the anterior and posterior circulation in the very low‐frequency (VLF) range during 4 h of sitting. All values are shown as the mean ± SD or median (interquartile range). ^*^ *P* < 0.05 versus base.

The present study is the first to investigate the impact of prolonged sitting on the dCA response in both the posterior (i.e., PCA) and the anterior (i.e., MCA) cerebral circulations by analysing spontaneous fluctuations in MAP and CBV. The findings reveal that 4 h of sitting decreased MCA *V*_m_, but decreased VLF nGain in the MCA, indicating an improvement in dCA rather than attenuation (Figures 1 and 2). In addition, PCA *V*_m_ and dCA in the PCA remained unchanged. The main findings of the present study are as follows: (1) PCA *V*_m_ was maintained during prolonged sitting despite a decrease in MCA *V*_m_; and (2) prolonged sitting did not impair dCA in either PCA or MCA. These findings suggest that the effect of a single prolonged sitting session on CBF regulatory function appears to be minimal in healthy, young individuals.

### 4.1 Effect of prolonged sitting on anterior and posterior CBF velocity

Prolonged sitting elicited different CBV responses between the anterior (MCA *V*_m_) and posterior (PCA *V*_m_) cerebral circulation (Figure 1). Specifically, throughout the 4 h sitting period, PCA *V*_m_ remains unchanged, whereas MCA *V*_m_ gradually decreased, suggesting that the PCA circulation might have a greater robustness to prolonged sitting in regulating CBF homeostasis compared with MCA circulation. Blood flowing in the PCA supplies the brainstem, which houses cardiovascular and respiratory control centres, and the hippocampus, which plays a crucial role in memory storage and recall (Tambini & Davachi, 2013). This underscores the importance of proper blood flow regulation in the posterior cerebral circulation for maintaining life (Tatu et al., 1996) and quality of life (Giovagnoli & Avanzini, 2000). Based on these insights, it is physiologically plausible that, during a 4 h sitting period, CBF homeostasis in the PCA is maintained preferentially to the MCA. Similar to the effects of prolonged sitting, during hypoxia (Ogoh et al., 2013b) and orthostatic stress (Ogoh et al., 2015), anterior CBF was reduced, while posterior CBF was less affected. Therefore, it seems that the posterior cerebral circulation, which is crucial for maintaining life support (autonomic nervous system), might prioritize the protection of CBF homeostasis compared with the anterior cerebral circulation during severe physiological conditions, such as excessive prolonged sitting. However, the physiological mechanisms underlying the differential CBV response to prolonged sitting between the MCA and PCA circulations remain unclear. Nonetheless, there may be some potential explanations for the physiological mechanisms underlying the reduction in MCA *V*_m_ but not in PCA *V*_m_ during prolonged sitting. In the present study, prolonged sitting did not impair dCA in the MCA, despite a decrease in MCA *V*_m_. Therefore, this decrease in MCA *V*_m_ during prolonged sitting might not be attributable to impaired dCA. Another potential mechanism for the decrease in MCA *V*_m_ could be changes in CO_2_ levels, because it is well established that orthostatic stress decreases $P_{\mathrm{ET},CO_{2}}$ (Ogoh et al., 2013a). Indeed, it is well known that CBF is influenced by the partial pressure of CO_2_ in the blood (Skow et al., 2013). Notably, it has been reported that CBF regulation in the anterior cerebral circulation is more sensitive to changes in CO_2_ levels compared with the posterior cerebral circulation (Skow et al., 2013). This finding might help to explain the different responses to prolonged sitting observed between MCA *V*_m_ and PCA *V*_m_. However, in the present study, $P_{\mathrm{ET},CO_{2}}$ remained unchanged during prolonged sitting (Table 1). Therefore, the reduction in MCA *V*_m_ observed here is unlikely to be attributable to changes in circulating CO_2_ levels.

### 4.2 Effect of dCA in the anterior and posterior cerebral circulation

**FIGURE 3:**
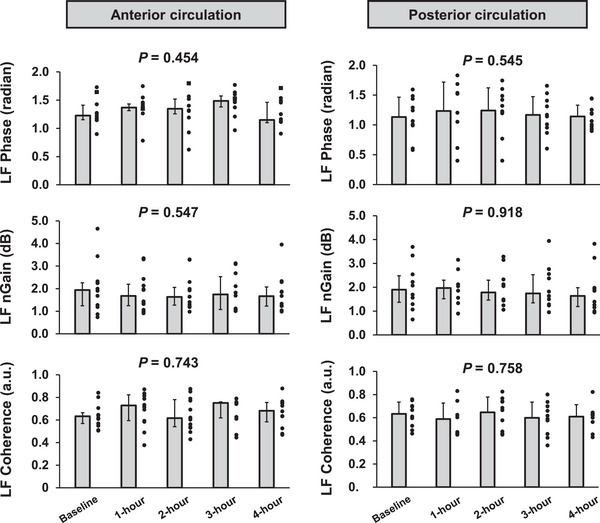
The transfer function phase, normalized gain (nGain) and coherence of the anterior and posterior circulation in the low‐frequency (LF) range during 4 h of sitting. All values are shown as the mean ± SD or median (interquartile range).

It is well known that dCA plays an important role in the regulation of CBF and that attenuation of dCA is related to an increased risk of cerebral disease (Chen et al., 2014; Dawson et al., 2003; den Abeelen et al., 2014; Xiong et al., 2017). Thus, in the present study, we focused on dCA to identify the mechanism of CBF regulation and understand cerebral circulation homeostasis following prolonged sitting. It has been suggested that dCA in the posterior cerebral circulation (e.g., dCA in the PCA) is more susceptible to orthostatic stress (Haubrich et al., 2004; Sato et al., 2012) and to sitting with eyes open (Nakagawa et al., 2009) compared with the anterior cerebral circulation (e.g., dCA in the MCA). Therefore, it is believed that dCA in the posterior cerebral circulation is more fragile than that in the anterior cerebral circulation (Haubrich et al., 2004; Sato et al., 2012; Washio et al., 2018). Based on these findings, we hypothesized that prolonged sitting affects dCA in the PCA more than that in the MCA and that dCA of the PCA would be attenuated during prolonged sitting. Contrary to our expectations, however, in the present study prolonged sitting resulted in a decrease in VLF nGain of the MCA, indicating an improvement in dCA in MCA, while other dCA indices of the MCA and PCA remained unchanged (Figures 2 and 3). Thus, dCA in both the MCA and PCA might be well maintained throughout the 4 h sitting period. The data of dCA in the PCA presented here are novel, whereas some studies (Carter et al., 2018, 2021; Maasakkers et al., 2020) have investigated the effect of prolonged sitting on dCA in the MCA, but these findings are inconsistent. Two previous studies (Carter et al., 2018; Maasakkers et al., 2020) have investigated dCA in the MCA, and their findings suggest that dCA indices of the MCA (i.e., phase and nGain) are not impaired, which is consistent with the present findings. However, in contrast, one previous study (Carter et al., 2021) indicates that dCA in the MCA is impaired following prolonged sitting. The reason for the inconsistent results regarding dCA in the MCA between the present study and previous studies (Carter et al., 2021) is unclear. However, the observed differences might be attributable to variations in experimental protocols. Notably, Carter et al. (2021) used a longer sitting duration (6 h) than the 4 h in the present study. Although both studies observed a similar reduction in MCA *V*_m_, with a decrease of 11% in the present study and 6% in the study by Carter et al. (2021), there were differences in cardiovascular responses, such as reductions in HR and MAP, in the latter. Additionally, prolonged sitting is likely to increase discomfort over time (Carter et al., 2021). Therefore, differences in experimental conditions, such as sitting duration and the resulting cardiovascular and psychological responses, might explain the inconsistent dCA response findings between the previous studies and the present study.

The dCA in the PCA was well maintained during 4 h of sitting. In contrast, the dCA in the MCA (VLF nGain decreased) improved rather than attenuated. The mechanism behind the unexpected result for the dCA in the MCA remains unclear. Regarding the slight differences in dCA response between MCA and PCA, it can be inferred that these differences were attributed to anatomical and physiological factors from previous studies. MCA and PCA, it can be inferred that these differences were attributed to anatomical and physiological factors from previous studies. (Edvinsson et al., 1976; Skow et al., 2013). For example, an earlier study (Aaslid et al., 1989) demonstrated that a hypocapnia‐induced increase in cerebrovascular resistance enhances dCA, indicating the possibility that mechanical change in cerebral vasculature could modify dCA. A difference in CBF response to changes in CO_2_ (cerebrovascular CO_2_ reactivity) between the anterior and posterior cerebral circulation was observed (Skow et al., 2013), and this difference in cerebrovascular CO_2_ reactivity between cerebral arteries can lead to variations in cerebral vascular resistance and, subsequently, dCA (Aaslid et al., 1989). However, in the present study, $P_{\mathrm{ET},CO_{2}}$ remained unchanged during 4 h of sitting (Table 1). Moreover, a previous study (Carter et al., 2018) also demonstrated that prolonged sitting did not alter cerebrovascular CO_2_ reactivity. Another possible factor that could induce different dCA responses is the varying influence of sympathetic activation on cerebral vasculature between the anterior and posterior cerebral arteries. It has been reported that sympathetic activation influences dCA, specifically, sympathetic activation enhances dCA throughout its effect on cerebral vascular tone (Edvinsson et al., 1976; Zhang et al., 2007). Given that the PCA has less sympathetic innervation than the MCA (Edvinsson et al., 1976), prolonged sitting‐induced sympathetic activation (Tamiya et al., 2024) might affect dCA differently between the MCA and PCA. However, in the present study, HR and MAP did not change from baseline to 4 h of sitting (Table 1), suggesting that the change in sympathetic activation might have been minimal during the 4 h sitting period. A previous study also reported no differences in dCA responses between the MCA and PCA, even during sympathetic activation induced by the cold pressor test, despite extremely large sympatho‐excitation (Washio et al., 2020). With these backgrounds, these physiological factors influencing dCA, physiological and anatomical differences (e.g., differences in diameter and sympathetic innervation), might not have been altered sufficiently to affect CBF regulation in both the anterior and posterior cerebral circulation during prolonged sitting. Thus, the mechanism of the slightly different dCA responses to prolonged sitting between the anterior and posterior cerebral circulation remains unknown and might be attributable to other factors. However, it is important to note that dCA in both the anterior and posterior cerebral circulation was well maintained during prolonged sitting.

Prolonged sitting can reduce the effects of the muscle pump and decrease venous return and central blood volume (Horiuchi & Stoner, 2021), potentially leading to fluid shifts. dCA is indeed influenced by rapid fluid shifts resulting from orthostatic stress (e.g., lower‐body negative pressure or head‐up tilt) (Sato et al., 2012; Zhang et al., 1998b). However, given that the cardiovascular response remained unchanged during the 4 h sitting period compared with the baseline in the present study (Table 1), this suggests that the fluid shift during prolonged sitting might not have been sufficient to affect dCA.

Cerebrovascular diseases, such as dementia and stroke, are widely recognized to impair dCA strongly (Chen et al., 2014; Dawson et al., 2003; den Abeelen et al., 2014; Xiong et al., 2017). However, in the present study, prolonged sitting did not attenuate dCA in either the MCA or the PCA (Figures 2 and 3). Interestingly, several recent studies have reported that patients with these cerebral diseases have either maintained (de Heus et al., 2018; Heutz et al., 2023; Reinhard et al., 2005) or even improved (Heutz et al., 2023; Weijs et al., 2023) dCA in comparison to healthy subjects. These findings suggest that dCA impairment might not be a fundamental factor in the increased risk of cerebral disease, and the role of dCA in brain health remains controversial. Nevertheless, the findings from our study suggest that the dCA response to acute prolonged sitting might not fully explain the increased brain dysfunction associated with a sedentary lifestyle (chronic prolonged sitting habits). Importantly, these findings pertain solely to the effects of acute prolonged sitting on cerebrovascular function and dynamics. Therefore, it remains uncertain whether chronic (repeated) prolonged sitting and the physiological changes associated with a sedentary lifestyle (e.g., weight gain and increased ABP) might impair dCA or elicit different responses in the MCA and PCA. To understand comprehensively the physiological mechanisms through which chronic prolonged sitting habits cause brain dysfunction, it is imperative to elucidate how the repeated episodes of prolonged sitting and associated physiological changes impact cerebrovascular functions, such as dCA and cerebrovascular dynamics, and how cardiovascular dynamics and function are involved. Thus, further investigation into these questions is needed.

### 4.3 Limitations

Some potential limitations of the present study should be considered. First, it is known that small changes in the MCA or PCA diameter, which transcranial Doppler cannot reflect, can influence MCA *V*_m_ or PCA *V*_m_. Indeed, severe conditions, such as extreme hypercapnia and rhythmic handgrip exercises, can lead to dilatation and constriction of the MCA, respectively (Verbree *et al.*, 2017, 2014). Therefore, the effect of prolonged sitting on MCA or PCA diameters is not clear, but when the blood flow remain constant, slight vaso‐dilatation and constriction can lead to changes in MCA *V*_m_ or PCA *V*_m_. Second, it is important to acknowledge that the study involved exclusively healthy young participants. In the present study, however, we aimed to evaluate the effect of prolonged sitting on cerebral circulation while isolating it from the influences of ageing. In addition, female participants were small sample size and exclusively in the early follicular phase. Consequently, the findings might not be generalizable to an ageing population or to females including other menstrual phases, although they suggested that dCA does not change throughout the menstrual cycle (Favre & Serrador, 2019). Third, no control conditions were implemented, such as incorporating intermittent breaks during the prolonged sitting period. Fourth, in the present study, TFA used spontaneous fluctuations in MAP and CBV to evaluate the dCA response over a 4 h sitting period. Therefore, we cannot discount the possibility of distinct regional differences in dCA when MAP and CBV are varied through perturbations (e.g., sit‐to‐stand protocol). Fifth, in the present study, we did not directly assess the stress associated with long‐duration experiments, such as bladder filling and overall participant comfort. However, we inquired verbally about any discomfort before the experiment and before each measurement session. If no discomfort was reported, the experiment proceeded. As a result, none of the 12 participants withdrew owing to discomfort. Therefore, we believe that stress effects related to bladder filling and overall comfort were minimal throughout the long‐duration experiment.

## 5 CONCLUSION

Contrary to our hypothesis, dCA in both the MCA and PCA was not impaired by acute prolonged sitting. This suggests that the response of CBF regulatory mechanisms to acute prolonged sitting does not account fully for the diminished brain function associated with a sedentary lifestyle (chronic prolonged sitting habits).

## AUTHOR CONTRIBUTIONS

Shotaro Saito and Shigehiko Ogoh conceptualized and designed the research; Shotaro Saito, Hayato Tsukamoto, Marino Karaki and Narumi Kunimatsu performed experiments; Shotaro Saito analysed data; Shotaro Saito and Shigehiko Ogoh interpreted the results of experiments; Shotaro Saito prepared figures; Shotaro Saito and Shigehiko Ogoh drafted the manuscript; all authors edited and revised the manuscript; and all authors approved the final version of the manuscript and agree to be accountable for all aspects of the work in ensuring that questions related to the accuracy or integrity of any part of the work are appropriately investigated and resolved. All persons designated as authors qualify for authorship, and all those who qualify for authorship are listed.

## CONFLICT OF INTEREST

None declared.

## Data Availability

The datasets generated and analysed in the present study are available from the corresponding author upon reasonable request.
